# Supplementary material for: Pat1 promotes processing body assembly by enhancing the phase separation of the DEAD-box ATPase Dhh1 and RNA
Source: eLife. 2019 Jan 16;8:e41415. doi: 10.7554/eLife.41415 (PMC6366900; doi:10.7554/eLife.41415)
Supplement: Supplementary file 1. [file elife-41415-supp1.docx]

**SUPPLEMENTARY TABLE S1**

**Yeast strains in this study**

| **Strain #** | **Genotype** |
| --- | --- |
| KWY165 | *MAT a ade2-1 can1-100 GAL phi+ his3-11,15 ura3-1 leu2-3, 112 trp1-1* |
| KWY276 | *MAT a ade2-1 can1-100 GAL phi+ his3-11,15 ura3-1 leu2-3, 112 trp1-1 dhh1::DHH1-GFP-HisMX6* |
| KWY1972 | *MAT a ade2-1 can1-100 GAL phi+ his3-11,15 ura3-1 leu2-3, 112 trp1-1 dcp2::DCP2-mCherry-NatMX6* |
| KWY1973 | *MAT a ade2-1 can1-100 GAL phi+ his3-11,15 ura3-1 leu2-3, 112 trp1-1 dhh1::DHH1-HisMX6 GFP dcp2::DCP2-mCherry-NatMX6* |
| KWY2226 | *MAT a ade2-1 can1-100 GAL phi+ his3-11,15 ura3-1 leu2-3, 112 trp1-1 pat1∆::KanMX6* |
| KWY3238 | *MAT alpha ade2-1 can1-100 GAL phi+ his3-11,15 ura3-1 leu2-3, 112 trp1-1 dhh1∆::KanMX6 dcp2::DCP2-mCherry-NatMX6 pDHH1-GFP-URA3* |
| KWY3239 | *MAT alpha ade2-1 can1-100 GAL phi+ his3-11,15 ura3-1 leu2-3, 112 trp1-1 dhh1∆::KanMX6 dcp2::DCP2-mCherry-NatMX6 pDHH1^DQAD^-GFP-URA3* |
| KWY3280 | *MAT a ade2-1 can1-100 GAL phi+ his3-11,15 ura3-1 leu2-3, 112 trp1-1 pat1::PAT1-GFP-HisMX6* |
| KWY4901 | *MAT alpha ade2-1 can1-100 GAL phi+ his3-11,15 ura3-1 leu2-3, 112 trp1-1 dhh1∆::KanMX6 dcp2::DCP2-mCherry-NatMX6 pDHH1^5X-Not^-GFP-URA3* |
| KWY5157 | *MAT alpha ade2-1 can1-100 GAL phi+ his3-11,15 ura3-1 leu2-3, 112 trp1-1 dhh1∆::TRP dhh1∆::KanMX6* |
| KWY5221 | *MAT alpha ade2-1 can1-100 GAL phi+ his3-11,15 ura3-1 leu2-3, 112 trp1-1 dhh1∆::TRP pat1∆::KanMX6 pDHH1 GFP-URA3* |
| KWY5224 | *MAT alpha ade2-1 can1-100 GAL phi+ his3-11,15 ura3-1 leu2-3, 112 trp1-1 dhh1∆::TRP pat1∆::KanMX6 pDHH1^DQAD^-GFP-URA3* |
| KWY5227 | *MAT alpha ade2-1 can1-100 GAL phi+ his3-11,15 ura3-1 leu2-3, 112 trp1-1 dhh1∆::TRP pat1∆::KanMX6 pDHH1^RNA3X^-GFP-URA3* |
| KWY5230 | *MAT alpha ade2-1 can1-100 GAL phi+ his3-11,15 ura3-1 leu2-3, 112 trp1-1 dhh1∆::TRP pat1∆::KanMX6 pDHH1^DQAD/RNA3X^-GFP-URA3* |
| KWY5601 | *MAT a ade2-1 can1-100 GAL phi+ his3-11,15 ura3-1 leu2-3, 112 trp1-1 pat1:: PAT1-HisMX6 dhh1::DHH1 GFP-URA3* |
| KWY5603 | *MAT a ade2-1 can1-100 GAL phi+ his3-11,15 ura3-1 leu2-3, 112 trp1-1 pat1::PAT1 AA::HisMX6 dhh1::DHH1 GFP-URA3* |
| KWY5609 | *MAT a ade2-1 can1-100 GAL phi+ his3-11,15 ura3-1 leu2-3, 112 trp1-1 pat1::PAT1 EE-HisMX6 dhh1::DHH1 GFP-URA3* |
| KWY5915 | *MAT alpha ade2-1 can1-100 GAL phi+ his3-11,15 ura3-1 leu2-3, 112 trp1-1 dhh1∆::TRP pat1∆::KanMX6 edc3::EDC3-mcherry:: HisMX6 pDHH1 GFP-URA3* |
| KWY5917 | *MAT alpha ade2-1 can1-100 GAL phi+ his3-11,15 ura3-1 leu2-3, 112 trp1-1 dhh1∆::TRP pat1∆::KanMX6 edc3::EDC3-mcherry:: HisMX6 pDHH1^DQAD^-GFP-URA3* |
| KWY5933 | *MAT alpha ade2-1 can1-100 GAL phi+ his3-11,15 ura3-1 leu2-3, 112 trp1-1 dhh1∆::TRP pat1∆::KanMX6 dcp2::DCP2-mcherry:: HisMX6 pDHH1 GFP-URA3* |
| KWY5935 | *MAT alpha ade2-1 can1-100 GAL phi+ his3-11,15 ura3-1 leu2-3, 112 trp1-1 dhh1∆::TRP pat1∆::KanMX6 dcp2::DCP2-mcherry:: HisMX6 pDHH1^DQAD^-GFP-URA3* |
| KWY5943 | *MAT alpha ade2-1 can1-100 GAL phi+ his3-11,15 ura3-1 leu2-3, 112 trp1-1 pat1:: PAT14A-HisMX6* |
| KWY5978 | *MAT alpha ade2-1 can1-100 GAL phi+ his3-11,15 ura3-1 leu2-3, 112 trp1-1 pat1:: PAT1-HisMX6 dhh1::DHH1 GFP-URA3 pGAL-PAT1-3XV5-LEU2* |
| KWY5980 | *MAT alpha ade2-1 can1-100 GAL phi+ his3-11,15 ura3-1 leu2-3, 112 trp1-1 pat1:: PAT1-HisMX6 dhh1::DHH1 GFP-URA3 pGAL-PAT1AA-3XV5-LEU2* |
| KWY5983 | *MAT alpha ade2-1 can1-100 GAL phi+ his3-11,15 ura3-1 leu2-3, 112 trp1-1 pat1:: PAT1-HisMX6 dhh1::DHH1 GFP-URA3 pGAL-PAT1 EE-3XV5-LEU2* |
| KWY6003 | *MAT alpha ade2-1 can1-100 GAL phi+ his3-11,15 ura3-1 leu2-3, 112 trp1-1 pat1Δ::KanMX6 dhh1Δ::TRP pDHH1^5X-Not^-GFP-URA3* |
| KWY6056 | *MAT alpha ade2-1 can1-100 GAL phi+ his3-11,15 ura3-1 leu2-3, 112 trp1-1 pat1::PAT14A-HismX6 dhh1Δ::TRP pDHH1^DQAD^-GFP-URA3* |
| KWY6058 | *MAT alpha ade2-1 can1-100 GAL phi+ his3-11,15 ura3-1 leu2-3, 112 trp1-1 pat1::PAT14A-HismX6 dhh1Δ::TRP pDHH1-GFP-URA3* |
| KWY6180 | *MAT alpha ade2-1 can1-100 GAL phi+ his3-11,15 ura3-1 leu2-3, 112 trp1-1 pGAL-PAT1 3X HA::KanMX6 dhh1::DHH1 GFP::URA3* |
| KWY6279 | *MAT a ade2-1 can1-100 GAL phi+ his3-11,15 ura3-1 leu2-3, 112 trp1-1 pat1::PAT1-HisMX6 edc3::EDC3 GFP-URA3* |
| KWY6403 | *MAT alpha ade2-1 can1-100 GAL phi+ his3-11,15 ura3-1 leu2-3, 112 trp1-1 pat1∆::pGAL-3X HA::KanMX6 dhh1::DHH1 GFP URA3* |
| KWY6467 | *MAT a ade2-1 can1-100 GAL phi+ his3-11,15 ura3-1 leu2-3, 112 trp1-1 pat1::PAT1AA-HisMX6 edc3::EDC3 GFP-URA3* |
| KWY6469 | *MAT a ade2-1 can1-100 GAL phi+ his3-11,15 ura3-1 leu2-3, 112 trp1-1 pat1::PAT1AA-HisMX6 dcp2::DCP2 GFP-URA3* |
| KWY6471 | *MAT a ade2-1 can1-100 GAL phi+ his3-11,15 ura3-1 leu2-3, 112 trp1-1 pat1::PAT1-HisMX6 dcp2::DCp2 GFP-URA3* |
| KWY6478 | *MAT a ade2-1 can1-100 GAL phi+ his3-11,15 ura3-1 leu2-3, 112 trp1-1 pat1::PAT1EE-HisMX6 dcp2::DCP2 GFP-URA3* |
| KWY6480 | *MAT a ade2-1 can1-100 GAL phi+ his3-11,15 ura3-1 leu2-3, 112 trp1-1 pat1::PAT1EE-HisMX6 edc3::EDC3 GFP-URA3* |
| KWY6544 | *MAT a ade2-1 can1-100 GAL phi+ his3-11,15 ura3-1 trp1-1 leu2-3, 112∆::CG-LEU2-NOT1^9X-Dhh1^-TAP not1∆::TRP1 dcp2::DCP2-mCherry-NatMX6* |
| KWY6604 | *MAT alpha ade2-1 can1-100 GAL phi+ his3-11,15 ura3-1 leu2-3, 112 trp1-1 pat1∆::TRP dhh1::DHH1 GFP URA3* |
| KWY6637 | *MAT alpha ade2-1 can1-100 GAL phi+ his3-11,15 ura3-1 leu2-3, 112 trp1-1 dhh1::DHH1 GFP-URA3 dcp2::DCP2-mCherry-NatMX6 pGAL-PAT1 AA-3XV5-LEU2* |
| KWY6643 | *MAT alpha ade2-1 can1-100 GAL phi+ his3-11,15 ura3-1 leu2-3, 112 trp1-1 pGAL-PAT1-3X HA::KanmX6 dhh1::DHH1 GFP: URA3 dcp2::DCP2-mCherry-NatMX6* |
| KWY6692 | *MAT alpha ade2-1 can1-100 GAL phi+ his3-11,15 ura3-1 leu2-3, 112 trp1-1 pGAL-PAT1 EE-3X-HA::KanMX6 dhh1::DHH1 GFP::URA3 dcp2::DCP2-mCherry-NatMX6* |
| KWY6836 | *MAT alpha ade2-1 can1-100 GAL phi+ his3-11,15 ura3-1 leu2-3, 112 trp1-1 pGAL-PAT1 3XHA::URA3 dhh1∆::KanMX6 edc3::EDC3 GFP-HisMX6 dcp2::DCP2-mCherry-NatMX6* |
| KWY7370 | *MAT a ade2-1 can1-100 GAL phi+ his3-11,15 ura3-1 leu2-3, 112 trp1-1 pat1::p-GAL- PAT1 NC-3X-HA-HYG dhh1::DHH1 GFP-URA3* |
| KWY7420 | *MAT alpha ade2-1 can1-100 GAL phi+ his3-11,15 ura3-1 leu2-3, 112 trp1-1 pGAL-PAT1 3XHA::KanMX6 dhh1::DHH1 RNA3X GFP-HismX6* |
| KWY7481 | *MAT a ade2-1 can1-100 GAL phi+ his3-11,15 ura3-1 leu2-3, 112 trp1-1 pat1::PAT14A GFP-URA3* |
| KWY7484 | *MAT a ade2-1 can1-100 GAL phi+ his3-11,15 ura3-1 leu2-3, 112 trp1-1 pat1::PAT1 NC-HYG dhh1::DHH1 GFP-HisMX6 dcp2::DCP2-mCherry-NatMX6* |
| KWY7554 | *MAT a ade2-1 can1-100 GAL phi+ his3-11,15 ura3-1 leu2-3, 112 trp1-1 pat1::PAT14A GFP-URA3 dcp2::DCP2 mcherry-KanMX6* |
| KWY7557 | *MAT a ade2-1 can1-100 GAL phi+ his3-11,15 ura3-1 leu2-3, 112 trp1-1 pat1::PAT1N-C GFP-HismX6* |
| KWY7562 | *MAT a ade2-1 can1-100 GAL phi+ his3-11,15 ura3-1 leu2-3, 112 trp1-1 pat1::PAT1N-C GFP-HisMX6 dcp2::DCP2 mcherry-NatMX6* |
| KWY7890 | *MAT a ade2-1 can1-100 GAL phi+ his3-11,15 ura3-1 leu2-3, 112 trp1-1* dhh1::dhh1^DQAD^-GFP-HisMX6 dcp2::DCP2-mCherry-NatMX6 |
| KWY8378 | *MAT a ade2-1 can1-100 GAL phi+ his3-11,15 ura3-1 trp1-1 leu2-3, 112∆::CG-LEU2-NOT1^9X-Dhh1^-TAP not1∆::TRP1 pat1::PAT1AA-HisMX6 dcp2::DCP2-mCherry-NatMX6* |
| KWY8622 | *MAT a ade2-1 can1-100 GAL phi+ his3-11,15 ura3-1 leu2-3, 112 trp1-1 edc3::NatMX6 scd6::KanMX6* |
| KWY8624 | *MAT a ade2-1 can1-100 GAL phi+ his3-11,15 ura3-1 leu2-3, 112 trp1-1 pat1:: HISMX6 edc3::NatMX6 scd6::KanMX6* |
| KWY8633 | *MAT a ade2-1 can1-100 GAL phi+ his3-11,15 ura3-1 leu2-3, 112 trp1-1 dhh1:: dhh1^R295D^ -GFP-HisMX6 dcp2::DCP2-mCherry-NatMX6* |
| KWY8638 | *MAT a ade2-1 can1-100 GAL phi+ his3-11,15 ura3-1 leu2-3, 112 trp1-1 dhh1:: dhh1^S292DN294D^-GFP-HisMX6 dcp2::DCP2-mCherry-NatMX6* |
| KWY8753 | *MAT a ade2-1 can1-100 GAL phi+ his3-11,15 ura3-1 leu2-3, 112 trp1-1 edc3::NatMX6 scd6::KanMX6 pat1::PAT1-NC-GFP::HisMX6* |
