# Supplementary material for: Pat1 promotes processing body assembly by enhancing the phase separation of the DEAD-box ATPase Dhh1 and RNA
Source: eLife. 2019 Jan 16;8:e41415. doi: 10.7554/eLife.41415 (PMC6366900; doi:10.7554/eLife.41415)
Supplement: Supplementary file 2. — Supplementary table S2A contains all the plasmids used in this study. Table Supplementary S2B contains the mutants used in this study and Table S2C describes the entire DNA oligos used for this manuscript. [file elife-41415-supp2.docx]

**SUPPLEMENTARY TABLE S2**

**A) Plasmids used in this study**

| **Plasmid #** | **Description** |
| --- | --- |
| pKW2312 | pRS316 P(DHH1)-DHH1-GFP |
| pKW2313 | pRS316 P(DHH1)-DHH1^DQAD^-GFP |
| pKW2867 | pRS316 P(DHH1)-DHH1^3X-RNA^-GFP |
| pKW3040 | pRS316 P(DHH1)-DHH1^DQAD/3X-RNA^-GFP |
| pKW3333 | pRS316 P(DHH1)-DHH1^5X-Not^-GFP |
| pKW3477 | pRS316 P(DHH1)-DHH1^DQAD/3X-RNA^-6xHis-PP7CP |
| pKW3570 | pFA6a-yEGFP-HIS3MX |
| pKW3756 | pNH605-PGAL-Pat1 WT (XbaI)-3X V5 |
| pKW3757 | pNH605-PGAL- Pat1^AA^ (XbaI)-3X V5 |
| pKW3758 | pNH605-PGAL- Pat1^EE^ (XbaI)-3X V5 |
| pKW3631 | pETMCN_HisV5_Dhh1-mCherry |
| pKW3688 | pETMCN_His_Dhh1-mCherry |
| pKW3641 | pETMCN_HisV5_Pat1-C |
| pKW3702 | pETMCN_HisV5_ Pat1-C^EE^ |
| pKW3920 | pETMCN_HisTEVGST3C_Pat1-N [5-79] |
| pKW4225 | pETMCN_GST-Pat1-NC-mCherry |
| pKW4356 | pETMCN_GST-Pat1-NC-GFP |
| pKW4357 | pETMCN_GST- Pat1-NC^4A-Dhh1^-GFP |
| pKW4358 | pETMCN_GST- Pat1-NC^EE^-GFP |

**B) Dhh1, Pat1 and Not1 mutants used in this study**

| **Mutant Protein** | **Mutation(s)** |
| --- | --- |
| Dhh1^DQAD^ | E195Q |
| Dhh1^Q-motif^ | F66R, Q73A |
| Dhh1^3X-RNA^ | R322A, S340A, R370A |
| Dhh1^5X-Not^ | R55E, F62E, Q282E, N284E, R335E |
| Dhh1^DQAD/3X-RNA^ | E195Q, R322A, S340A, R370A |
| Dhh1^5X-Not/3X-RNA^ | R55E, F62E, Q282E, N284E, R322A, R335E, S340A, R370A |
| Dhh1^R295D^ (Mut3B) | R295D |
| Dhh1^S292DN294D^ (Mut3A) | S292D, N294D |
| Not1^9X-Dhh1^ | F791A, N795A, K804A, E823R, N834A, Y835A, K962A, F967A, E970A |
| Pat1^AA^ | S456A S457A |
| Pat1^EE^ | S456E S457E |
| Pat1^4A-Dhh1^ | D39A E40A T41A F42A |
| Pat1-N | Pat1-N (5-79) |
| Pat1-N-C | Pat1 N (5-79)- (GGS)4- C- (456-787) |

**C) Oligos used in this study**

| **Oligo #** | **Sequence** | **Description** |
| --- | --- | --- |
| CH393 | tgttcattattcatcatggaccaagccgataaaatgttatctcgt | DHH1, fw; mutagenic primer E195Q for Dhh1^DQAD^ mutant |
| CH394 | acgagataacattttatcggcttggtccatgatgaataatgaaca | DHH1, rev; mutagenic primer E195Q for Dhh1^DQAD^ mutant |
| CH574 | atggtttttcaaaaccggcttcttcaatacccattaacagctctcttttcaaataaaaatc | DHH1, rev; mutagenic primer F62E for Dhh1^5X-Not^ mutant |
| CH575 | cttaaatacaaagggtaatacttttgaagatttttatttgaaagaagagctgttaatgggtattttt | DHH1, fw; mutagenic primer R55E for Dhh1^5X-Not^ mutant |
| CH576 | gatttttatttgaaaagagagctgttaatgggtattgaagaagccggttttgaaaaaccat | DHH1, fw; mutagenic primer F62E for Dhh1^5X-Not^ mutant |
| CH577 | aaaaatacccattaacagctcttctttcaaataaaaatcttcaaaagtattaccctttgtatttaag | DHH1, rev; mutagenic primer R55E for Dhh1^5X-Not^ mutant |
| CH578 | cacggttagtagaattacaaaaaataatggcttgctcaatttcaagcttagagaataaagtatttaaacaatgtagc | DHH1, rev; mutagenic primer Q282E and N284E for Dhh1^5X-Not^ mutant |
| CH579 | gctacattgtttaaatactttattctctaagcttgaaattgagcaagccattattttttgtaattctactaaccgtg | DHH1, fw; mutagenic primer Q282E and N284E for Dhh1^5X-Not^ mutant |
| CH580 | atcggagcagaccaatgtttcaaccttaccttgacgaaattcgtgg | DHH1, rev; mutagenic primer R335E for Dhh1^5X-Not^ mutant |
| CH581 | ccacgaatttcgtcaaggtaaggttgaaacattggtctgctccgat | DHH1, fw; mutagenic primer R335E for Dhh1^5X-Not^ mutant |
| Ch849 | atcaaattcccctcttctgatgcttaattgccccgattgcagcctgttattgatactatt | For stitch PCRs to make Pat1 AA and EE, pringle fw |
| CH853 | taaaaaataagggagaaaaaaaaatacatgcgtaagtacattaaaattacaggaaaaatc gaattcgagctcgtttaaac | For stitch PCRs to make Pat1 AA and EE , pringle rev |
| CH854 | attaccgttgttgaacgcgtaagcagctcttcttctttgcttaga | For stitch PCRs to make Pat1 AA, rev |
| CH855 | tctaagcaaagaagaagagctgcttacgcgttcaacaacggtaat | For stitch PCRs to make Pat1 AA, fw |
| CH856 | attaccgttgttgaacgcgtattcttctcttcttctttgcttaga | For stitch PCRs to make Pat1 EE, rev |
| CH857 | tct aag caa aga aga aga gaa gaa tac gcg ttc aac aac ggt aat | For stitch PCRs to make Pat1 EE, fw |
| CH858 | ttaattaacccggggatccg ttactttagttctgatatttcaccatcgcgatacaccaaccccataac | For stitch PCRs to make Pat1 EE, rev |
| CH944 | aacttcttcaagtcctgcttgtcccaggagggttcaggga | For stitch PCRs to make Pat1 4A GFP tagged, fw |
| CH945 | aac ctg aac att atc acc agc agc agc agc gttcaaatagtcgttctc | For stitch PCRs to make Pat1 4A GFP tagged, rev |
| CH946 | gaacgactatttgaac gct gct gct gct ggtgataatgttcaggttgg | For stitch PCRs to make Pat1 4A GFP tagged, fw |
| CH1035 | tttcatggcgatgccacctggtcagtcacaaccccagtat cggatccccgggttaattaa | Dhh1, fw; C-terminal GFP tagging |
| CH1036 | gcgtatctcaccacagtagttattttttcttagatattct gaattcgagctcgtttaaac | Dhh1 rev; C-terminal GFP tagging |
| CH1037 | ttcagggtctaatgaattattaagcattttgcataggaag cggatccccgggttaattaa | Dcp2, fw; C-terminal mcherry tagging |
| CH1038 | catttacagtgtgtctataaaacgtataacacttattcttgaattcgagctcgtttaaac | Dcp2, rev; C-terminal mcherry tagging |
| CH1039 | tgatcttttcgtcactgacgggtccctgctattagatttgcggatccccgggttaattaa | Edc3, C- terminal mcherry, fw |
| CH1040 | tatacgtatgtatccagtttaggctaaagtaattcttggtgaattcgagctcgtttaaac | Edc3, C- terminal mcherry, rev |
| CH1116 | gaggagaacgactatttgaac gct gct gct gct ggtgataatgttcaggtt | For stitch PCRs to make Pat1 4A GFP tagged, fw (pringle) |
| CH1117 | aacctgaacattatcacc agc agc agc agc gttcaaatagtcgttctcctc | For stitch PCRs to make Pat1 4A GFP tagged, rev (pringle) |
| CH1550 | ttccccattttatcaaaaatcatcctgccaccattttgtccaaggatcgcactatcgccc gaattcgagctcgtttaaac | Pgal-Pat1 tagging: fw |
| CH1551 | ttcaaagtccagaggaccatcccgcgcattaccgctattttctaacccaaagaaggacat gcactgagcagcgtaatctg | Pgal-Pat1 tagging: rev |
| CH1768 | aaaataagggagaaaaaaaaatacatgcgtaagtacattaaaattacaggaaaaatctta gcactgagcagcgtaatctg | K/O Pat1 in Pgal-3HA-Pat1 : rev |
| CH1879 | aaggttttaaccggaagtaagagcagcaagaagcactagcagaacgcggccgccagctgaa | Pat1, fw; C-terminal GFP tagging |
| CH1878 | tacatgcgtaagtacattaaaattacaggaaaaatcttatgaattcgagctcgtttaaac | Pat1, rev; C-terminal GFP tagging |
| UC1943 | ctcttgcgttactatggtggtattattgatgctaatacgggcgcataggccactagtggatc | For pat1 delete, rev |
| UC1942 | aaggttttaaccggaagtaagagcagcaagaagcactagcagaacgcggccgccagctgaa | For pat1 delete,fw |
| CH2394 | ttccccattttatcaaaaatcatcctgccaccattttgtccaaggatcgcactatcgccctcactatagggagaccggca | Pgal-Pat1 tagging: fw |
| CH2395 | ttcaaagtccagaggaccatcccgcgcattaccgctattttctaacccaaagaaggacat cattttgagatccgggtttt | Pgal-Pat1 tagging: rev |
| CH2762 | gggttagaaaatagcggtaatgcgcgggatggtcctctgg | For stitch PCRs to make Pat1 NC, fw |
| CH2768 | tcgcttatttagaagtggcgcgttacttaagctcgagcataacgtttaggaa | For stitch PCRs to make Pat1 NC, rev |
| CH2769 | cgttatgctcgagcttaagtaacgcgccacttctaaataagcgaatttctta | For stitch PCRs to make Pat1 NC, fw |
| CH2790 | ttaaagccttcgagcgtcccaaaaccttctcaagcaaggttttcagtataat | For stitch PCRs to make Pat1 NC, rev |
| CH2791 | taaaaaataagggagaaaaaaaaatacatgcgtaagtacattaaaattacaggaaaaatcttaaagccttcgagcgtcccaa | For stitch PCRs to make Pat1 NC, rev |
| CH2766 | gacgtcccggactatgcaggatcctatccatatgacgttccagattacgctgctcagtgcgggttagaaaatagcggtaa | For Pat1 NC HYG (pgal OE), fw |
| CH3005 | gtacaccttgaaggaagcaaaggttttaaccggaagtaagagcagcaagaagcactagca gggttagaaaatagcggtaa | For stitch PCRs to make Pat1 NC, fw |
| CH3054 | gggttagaaaatagcggtaatgcgcgggatggtcctctgg | For stitch PCRs to make Pat1 NC GFP tagged, fw |
| CH3060 | catgttaattaacccggggatccgcttaagctcgagcataacgtttaggaac | For stitch PCRs to make Pat1 NC GFP tagged, rev |
| CH3061 | gttcctaaacgttatgctcgagcttaagcggatccccgggttaattaacatg | For stitch PCRs to make Pat1 NC GFP tagged, fw |
| Ch3062 | gaattcgagctcgtttaaactggatggcggcgttagtatcgaatcgacagca | For stitch PCRs to make Pat1 NC GFP tagged, rev |
| CH3058 | gtacaccttgaaggaagcaaaggttttaaccggaagtaagagcagcaagaagcactagcagggttagaaaatagcggtaa | For stitch PCRs to make Pat1 NC GFP tagged, fw (pringle) |
| CH3063 | taaaaaataagggagaaaaaaaaatacatgcgtaagtacattaaaattacaggaaaaatcgaattcgagctcgtttaaact | For stitch PCRs to make Pat1 NC GFP tagged, rev (pringle) |
| CH4227 | cgccattcgcataataacttatcccaggcctaaaatacgacaagaaagaaaatagtagtaatgggttccatcaataataa | For stitch PCRs to make Dhh1^S292DN294D^(Mut3A)-GFP and Dhh1^R295D^(Mut3A)-GFP tagged, fw (pringle) |
| CH4228 | ttttcttggctagtaattcgacatcgttagtagaattacaaaaaa | For stitch PCRs to make Dhh1^R295D^ Dhh1^R295D^(Mut3A)-GFP, Dhh1^R295D^(Mut3A)-GFP tagged, rev |
| CH4229 | ttttttgtaattctactaacgatgtcgaattactagccaagaaaa | For stitch PCRs to make Dhh1^S292DN294D^(Mut3A)-GFP and Dhh1^R295D^(Mut3A)-GFP tagged, rev |
| CH4231 | acgattagatcacaaaaaaagcgtatctcaccacagtagttattttttcttagatattctgaattcgagctcgtttaaac | For stitch PCRs to make Dhh1^S292DN294D^(Mut3A)-GFP, Dhh1^R295D^(Mut3A)-GFP tagged, rev (pringle) |
| CH4232 | ttttcttggctagtaattcgacacgatcagtatcattacaaaaaataatggcttgat | For stitch PCRs to make Dhh1^S292DN294D^(Mut3A)-GFP tagged, rev |
| CH4233 | atcaagccattattttttgtaatgatactgatcgt gtcgaattactagccaagaaaa | For stitch PCRs to make Dhh1^S292DN294D^(Mut3A)-GFP tagged, fw |
| CH4234 | ttaattaacccggggatccgatactggggttgtgactgaccaggtggcatcgccatgaaatgttc | For stitch PCRs to make Dhh1^S292DN294D^(Mut3A)-GFP and Dhh1^R295D^(Mut3A)-GFP tagged, rev |
| US5021 | ttcgtacattggtctgcgccgatttattgacccgt | DHH1, fw; mutagenic primer S340A for Dhh1^3X-RNA^ mutant |
| US5022 | acgggtcaataaatcggcgcagaccaatgtacgaa | DHH1, rev; mutagenic primer S340A for Dhh1^3X-RNA^ mutant |
| US5023 | ccaaaacggcagaaacatatttacatgctattggtagatccggtagg | DHH1, fw; mutagenic primer R370A for Dhh1^3X-RNA^ mutant |
| US5024 | cctaccggatctaccaatagcatgtaaatatgtttctgccgttttgg | DHH1, rev; mutagenic primer R370A for Dhh1^3X-RNA^ mutant |
| UC6097 | atcccaggcctaaaatacgacaagaaagaaaatagtagta*atgggttccatcaataataa* | DHH1; fw; For integrating *DHH1* mutants into *dhh1∆* strains |
| UC6098 | tatctcaccacagtagttattttttcttagatattct*ttaatactggggttgtgactgac* | DHH1; rv; For integrating *DHH1* mutants into *dhh1∆* strains |
